# Isolation and Characterization of Akhmeta Virus from Wild-Caught Rodents (*Apodemus* spp.) in Georgia

**DOI:** 10.1128/JVI.00966-19

**Published:** 2019-11-26

**Authors:** Jeffrey B. Doty, Giorgi Maghlakelidze, Irakli Sikharulidze, Shin-Lin Tu, Clint N. Morgan, Matthew R. Mauldin, Otar Parkadze, Natia Kartskhia, Maia Turmanidze, Audrey M. Matheny, Whitni Davidson, Shiyuyun Tang, Jinxin Gao, Yu Li, Chris Upton, Darin S. Carroll, Ginny L. Emerson, Yoshinori Nakazawa

**Affiliations:** a U.S. Centers for Disease Control and Prevention, Poxvirus and Rabies Branch, Atlanta, Georgia, USA; b U.S. Centers for Disease Control and Prevention, South Caucuses Office, Tbilisi, Georgia; c National Center for Disease Control and Public Health, Tbilisi, Georgia; d Department of Biochemistry and Microbiology, University of Victoria, Victoria, Canada; e National Food Agency, Tbilisi, Georgia; f Laboratory of the Ministry of Agriculture, Tbilisi, Georgia; University of Illinois at Urbana Champaign

**Keywords:** *Orthopoxvirus*, Akhmeta virus, poxvirus, viral isolation, rodents, *Apodemus*, Georgia, genetic recombination

## Abstract

In 2013, a novel orthopoxvirus was detected in skin lesions of two cattle herders from the Kakheti region of Georgia (country); this virus was named Akhmeta virus. Subsequent investigation of these cases revealed that small mammals in the area had serological evidence of orthopoxvirus infections, suggesting their involvement in the maintenance of these viruses in nature. In October 2015, we began a longitudinal study assessing the natural history of orthopoxviruses in Georgia. As part of this effort, we trapped small mammals near Akhmeta (*n* = 176) and Gudauri (*n* = 110). Here, we describe the isolation and molecular characterization of *Akhmeta virus* from lesion material and pooled heart and lung samples collected from five wood mice (Apodemus uralensis and Apodemus flavicollis) in these two locations. The genomes of Akhmeta virus obtained from rodents group into 2 clades: one clade represented by viruses isolated from *A. uralensis* samples, and one clade represented by viruses isolated from *A. flavicollis* samples. These genomes also display several presumptive recombination events for which gene truncation and identity have been examined.

**IMPORTANCE** Akhmeta virus is a unique *Orthopoxvirus* that was described in 2013 from the country of Georgia. This paper presents the first isolation of this virus from small mammal (Rodentia; *Apodemus* spp.) samples and the molecular characterization of those isolates. The identification of the virus in small mammals is an essential component to understanding the natural history of this virus and its transmission to human populations and could guide public health interventions in Georgia. Akhmeta virus genomes harbor evidence suggestive of recombination with a variety of other orthopoxviruses; this has implications for the evolution of orthopoxviruses, their ability to infect mammalian hosts, and their ability to adapt to novel host species.

## Abstract (precis)

Akhmeta virus is a unique *Orthopoxvirus* that was described in 2013 from the country of Georgia. This paper presents the first isolation of this virus from small mammal (Rodentia; *Apodemus* spp.) samples and the molecular characterization of those isolates. The identification of the virus in small mammals is an essential component to understanding the natural history of this virus and its transmission to human populations and could guide public health interventions in Georgia. Akhmeta virus genomes harbor evidence suggestive of recombination with a variety of other orthopoxviruses; this has implications for the evolution of orthopoxviruses, their ability to infect mammalian hosts, and their ability to adapt to novel host species.

## INTRODUCTION

*Akhmeta virus* (AKMV), a member of the genus *Orthopoxvirus* (OPXV), was first isolated in 2013 from lesion material collected from two cattle herders in the country of Georgia (1). These men presented with lesions on their hands that physicians suspected to be the result of cowpox virus (CPXV) infections. Samples examined by the National Center for Disease Control and Public Health (NCDC) in Tbilisi, Georgia, and the U.S. Centers for Disease Control and Prevention (CDC) in Atlanta, GA, USA, were found to contain a novel OPXV via viral isolation and DNA sequencing. An investigation into the potential source of the virus revealed that although the herder’s cattle did not present with signs of active infections (live virus), 100% (*n* = 11) of the serum samples collected from those animals were positive for anti-OPXV IgG antibodies (1). This investigation also collected samples from small mammals; although none of these samples contained OPXV DNA or live virus, 35.5% (*n* = 34) were positive for anti-OPXV IgG antibodies. This suggested that rodents and shrews in this region (*Apodemus* spp., 1/17 [5.9%]; *Dryomys*, 1/1 [100%]; *Microtus*, 7/11 [63.6%]; and *Sorex*, 3/3 [100%]) were likely regularly exposed to OPXVs. However, the assay used in that investigation was OPXV generic and could not distinguish between the different OPXV species.

Evidence of OPXV infection in rodents was previously reported in Georgia on two occasions in the 1980s from animals captured in the southeastern region of Kakheti. Tsanava et al. (2) reported isolating *Cowpox virus* from pooled tissue samples (*n* = 280) collected from red-tailed jirds (Meriones libycus) in 1986. The isolates were identified as cowpox virus based on pock morphology on chicken embryo chorioallantoic membranes (CAM), hemagglutinating activity, and pathogenicity in rabbits; however, these isolates are no longer available for further characterization. Rodents of the genera *Microtus* (*n* = 442) and *Apodemus* (*n* = 150) were also sampled during this collection, but no viral isolates were obtained from these specimens. Additionally, in a serosurvey of small mammals conducted in an area near where the cowpox isolate was obtained, anti-OPXV virus neutralizing antibodies (VNA) were detected in 9.3% (5/54) of red-tailed jirds and 1.8% (3/170) of roof rats (Rattus norvegicus) (3). Rodents of the genera *Microtus* (*n* = 280) and *Apodemus* (*n*, unknown) and shrews of the genus *Sorex* (*n* = 28) were found to be negative for VNA.

The various problems associated with surveying small mammals and the difficulties in isolating OPXVs from these samples have likely slowed the progress of identifying OPXV reservoirs (4). Cowpox-like viruses are more frequently isolated from wild rodents than other OPXVs. At least five isolates have been recovered from voles of the genus *Microtus* (5, 6) and rodents inhabiting arid climates of Eurasia, including yellow susliks (Spermophilus fulvus), big gerbils (Rhombomys opimus), and red-tailed jirds (*Meriones libycus*) (2, 7, 8). In addition, *Monkeypox virus*, *Vaccinia virus*, *Taterapox virus*, and *Volepox virus* have been isolated from wild rodents (9–12). In this paper, we detail the first detection, isolation, and characterization of Akhmeta virus in samples collected from wild rodents.

## RESULTS

**FIG 1:**
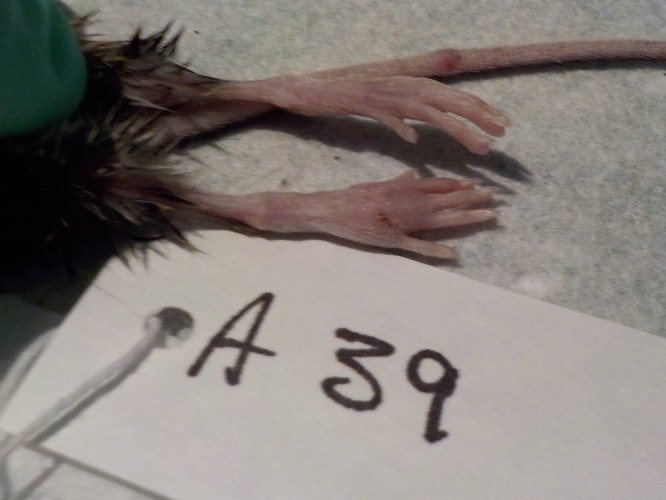
AKMV lesions on the foot and tail of a wild-caught rodent (*Apodemus flavicollis*).

A total of 286 small mammals (Table 1) were sampled from the two locations (*n* = 110, Gudauri; *n* = 176, Akhmeta). Of these animals, three from Gudauri (2 *Apodemus* and 1 *Microtus*) and six from Akhmeta (all *Apodemus*) had pox-like lesion samples collected in the field. The generic orthopox real-time PCR assay was first conducted on lesion material, and then all tissue samples were screened. In total, samples collected from 5 (1.7%) animals were found to be PCR positive (Table 2). In addition to lesion samples, heart plus lung, spleen plus kidney, and dried blood samples on Nobuto filter paper were found to be positive in at least one animal. All samples found to be positive with the generic OPXV assay were also positive by the AKMV-specific assay, indicating all positive animals examined here were infected with Akhmeta virus. Each animal with AKMV-positive lesions was found with two visible lesions, which were located on the foot (A39 and A40), nose (G66), and tail (A39, A40, and G66) (Fig. 1).

**TABLE 1:** Small mammals sampled in Gudauri and Akhmeta, Georgia, 2016

Genus	No. of animals		
	Gudauri (July)	Akhmeta (October)	Total
*Apodemus*	74	146	220
*Microtus*	18	28	46
*Sorex*	15	2	17
*Chionomys*	2	0	2
*Mustela*	1	0	1
			
Total	110	176	286

**TABLE 2:** Real-time PCR results from the generic orthopox assay and the Akhmeta virus-specific assay for small-mammal samples collected in Georgia, 2016

ID^*a*^	*C_t_* value or result^*b*^								
	Lesion		Liver		Spleen+kidney		Heart+lung		Nobuto
	OPXV	AKMV	OPXV	AKMV	OPXV	AKMV	OPXV	AKMV	AKMV
G66	32	28	Neg	Neg	Neg	Neg	Neg	36	Neg
A39	23	19	Neg	Neg	Neg	35	Inconcl	34	33
A40	24	20	Neg	Neg	Neg	Inconcl	Neg	Neg	Inconcl
G87	NA	NA	Neg	Neg	Neg	Neg	37	35	35
A76	NA	NA	Neg	Neg	Neg	Neg	37	36	32

a ID, identifier.

b *C_T_* values ranging from 38 to 40 were considered inconclusive (Inconcl). OPXV, orthopox virus; AKMV, Akhmeta virus; NA, not available.

**FIG 2:**
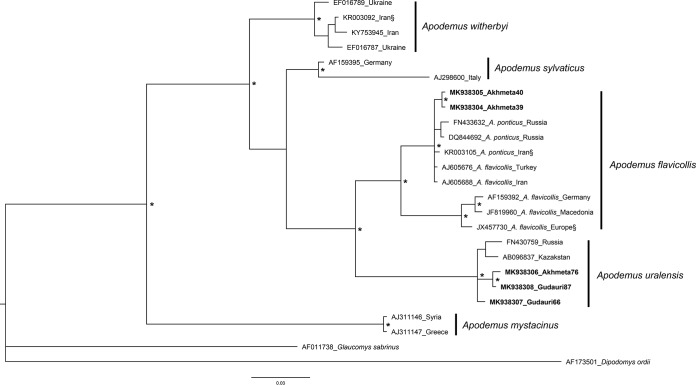
Phylogenetic tree based upon 409-bp fragments of the cytochrome *b* gene that shows the *Apodemus* rodents examined in this study along with reference sequences from other species of *Apodemus* known to occur in this region. Bayesian consensus tree based on two independent runs of 5 million generations each. *, node with >95 posterior probabilities; §, sequence with uncertain sampling localities. Vertical black bars show clades corresponding to known species.

Viral isolates were obtained from three PCR-positive lesion samples (A39, A40, and G66) as well as from three pooled PCR-positive heart plus lung samples (A39, A76, and G87). Viable virus was cultured from both the lesion and heart plus lung samples from one individual (A39). Both lesion and heart plus lung sample types were represented by two high-titer (10^4^ to 10^6^) samples and one low-titer (10^1^) sample (Table 3). Based on external morphology and cytochrome *b* sequence data (Fig. 2), the animals from which these samples were collected were identified as pygmy field mice (Apodemus uralensis; G66, G87, and A76) and yellow-necked mice (Apodemus flavicollis; A39 and A40). These cytochrome *b* sequences were deposited in GenBank (MK938304 to MK938308).

**TABLE 3:** Live virus titrations of AKMV PCR-positive rodent samples collected in Gudauri and Akhmeta, Georgia, 2016

ID^*a*^	Titer (PFU/ml of tissue homogenate)^*b*^			
	Lesion	Spleen+kidney	Heart+lung	Nobuto
G66	4.44 × 10^1^	NA	Neg	NA
A39	8.33 × 10^4^	Neg	1.11 × 10^1^	Neg
A40	1.02 × 10^5^	NA	NA	NA
G87	NA	NA	3.67 × 10^5^	NA
A76	NA	NA	1.23 × 10^6^	NA

a ID, identifier.

b NA, not available.

**FIG 3:**
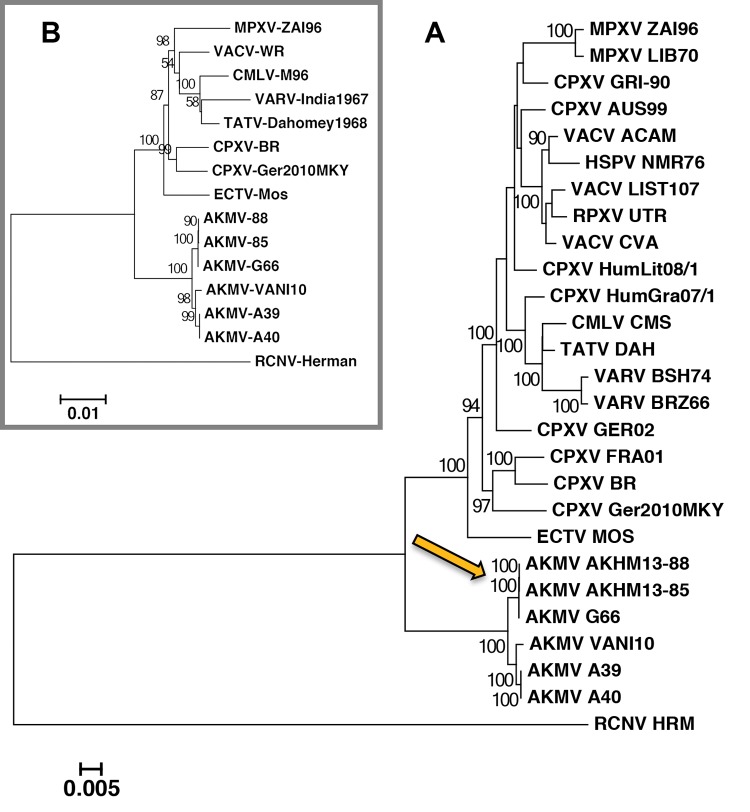
Maximum likelihood analysis examining Old World orthopoxviruses with raccoonpox virus as the outgroup taxon. Analysis examined relationship of Akhmeta virus (AKMV) isolates to each other as well as other congeners. (A) Nucleotide core tree; orange arrow indicates point in evolutionary history where large recombination event discussed in text (and highlighted in orange in Fig. 4, bottom) is hypothesized to have occurred. Bootstrap values less than 80 are not shown. (B) Conserved chordopoxvirus genes (amino acid) tree; reproduces the AKMVs position that branches off earlier than the OPXVs as seen in the nucleotide tree in panel A and shows CPXV-Ger2010MKY at its correct position adjacent to CPXV-BR.

The raw reads from the Illumina for the 3 sequenced AKMV isolates (G66, A39, and A40) yielded 2 to 3 contigs each with good average sequence depths (∼495× for A39, ∼1,056× for G66, and ∼1,678× for A40). Contigs and inverted terminal repeats (ITRs) were manually extended into full genomes and deposited in GenBank (G66, MN244296; A39, MN244297; A40, MN244298). Note that G66 genome has a 7-Ns placeholder at genome position 97727 to 97733. These Ns are situated within a repeat region consisting of a CTTATAT (7 nucleotide [nt]) motif that is repeated up to 18 times in AKMV reference strain 88. The genome assembler was unable to confidently resolve sequence at this region as the G66’s read length was only 106 nt long (A39 and A40 did not display this issue, as those reads were 126 nt in length). Attempts to perform PCR across the regions flanking the Ns were unsuccessful; however, G66 is 99.99% identical to AKMV-88, and calculation with the sequenced depths around this region supports a range around 16 to 18 repeats. These 3 new AKMV genomes were all closely related to those previously described from human cases (13). G66 is very similar to AKMV-88 and AKMV-85 isolates, while A39 and A40 are more similar to VANI10 (Fig. 3). Their 81 conserved chordopoxvirus genes on average have 99.5% amino acid (aa) identity (id) with reference AKMV-88, a higher level of identity than closely related sister species CPXV and vaccinia virus (VACV), which share 99.1%. All AKMVs harbor the same set of genes (no unique genes), although some have truncations (see Table S1 in the supplemental material). It is therefore suggested that the AKMVs examined here are different strains of the same virus species. A recent poxvirus phylogeny report showed CPXV-Ger2010MKY (LT896721) branches off earlier than all of the extant orthopoxviruses, at a position similar to that of Akhmeta viruses (14). This relationship was not reproduced in Fig. 3A (core nucleotide tree) nor in an amino acid tree using protein domains of conserved chordopoxvirus genes (Fig. 3B). Our phylogeny shows it branched adjacent to CPXV-BR; this is consistent with the first phylogeny reported for CPXV-Ger2010MKY (15).

### Genome comparison and visual summary.

Table S1 compares AKMV strains 85, G66, VANI10, A39, and A40 against reference AKMV-88 annotations and matches AKMV genes to orthologs in CPXV-BR, CPXV-GRI-90, and VACV-Cop references. Following the ascending numberings, one is able to visually assess missing and inserted genes. In summary, compared to reference strain CPXV-BR, AKMVs are missing 16 gene annotations (2 in the ITR), though only three have been characterized. CPXV-058, -096, -130, -170, -192, and -214 are small open reading frames (ORFs) overlapping larger ORFs on the other strand; these types of ORFs have a different isoelectric point (pI) and amino acid composition and are hypothesized as unlikely to represent real genes (16). AKMVs are also missing equivalent annotations of CPXV-160 and -161, which denote an uncharacterized ORF and the C-terminal fragmented ORF of P4c precursor (caused by frameshift), respectively. This is not eventful because, in contrast, AKMVs simply have 1 full-length P4c precursor gene instead of 2 fragments, and (for the same reason as the above) CPXV-160 is a small ORF situated on the opposite strand between the 2 fragments of the P4c precursor gene (CPXV-159 and -161) and is thus unlikely to be a real gene. CPXV-001, -002, -004, -063, and -216 are small hypothetical ORFs no larger than 72 codons. This leaves about 3 known genes that are missing in AKMVs: CPXV-013 (BTB kelch-domain containing protein), CPXV-218 (alpha or beta interferon [IFN-α/β] receptor glycoprotein), and CPXV-221 (tumor necrosis factor [TNF] receptor CrmD).

**FIG 4:**
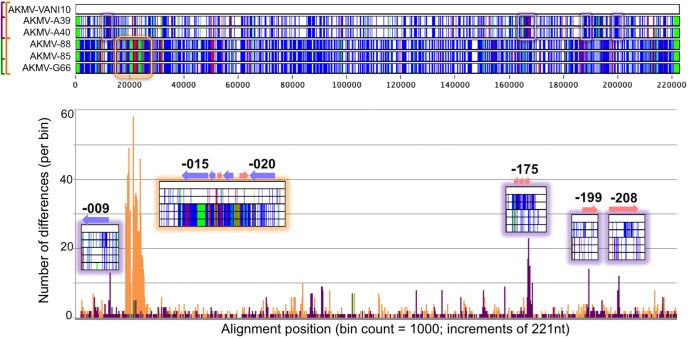
Nucleotide differences among AKMV isolates. (Top) BBB Visual Summary displaying SNPs from aligned AKMV genomes with respect to strain VANI10. Blue, red, and green lines indicate SNPs, insertions, and deletions, respectively. Boxes highlight regions with unusually high numbers of differences to strain VANI10. (Bottom) Bar chart of SNP distribution in 1,000 “bins” of 221 nucleotides along genomes. Genomes compared: orange, the 2 AKMV subclades; purple, VANI10 to A39 and A40; green, G66 to 88 and 85. Insets show zoomed-in Visual Summary blocks corresponding to peaks in the bottom panel; encoded AKMV genes are labeled in bold; genes transcribed to the left and right are noted as blue and pink arrows, respectively.

Table S1 also compares AKMV strains 85, G66, VANI10, A39, and A40 against reference AKMV-88 in terms of percent amino acid identity and protein sizes. The percent amino acid identities that are outside the standard deviation (SD) for that particular genome average are highlighted in red. For example, raw data show that for VANI10, the average gene percent amino acid identity to reference is 98.75% with an SD of 3.33%. Therefore, any values of <95.42% are highlighted in red. For A40 and A39, the average gene percent amino acid identities to reference are 98.56% and 98.58% with SDs of 3.57% and 3.52%, respectively; therefore, values need to be lower than 94.99% or 95.06% to be highlighted in red. Truncation and extension of genes (protein size difference) are dictated by percent size compared to the reference gene and highlighted in yellow if out of norm for each genome. Overall, the 6 AKMVs harbor the same set of genes with some size variations due to truncations (yellow highlighting in Table S1). By scanning for blocks of unusually lowered percent amino acid identities (red highlighting in Table S1), we discovered regions with unusually high single nucleotide polymorphism (SNP) density. Therefore, the distribution of SNPs among the genomes was examined in greater detail using the Base-By-Base sequence editor (BBB) (17). Figure 4 displays a number of sequence blocks that clearly have more SNPs than the general overall density found when AKMV-VANI10 is compared to both subclades of the AKMV genomes.

The large putative recombination event (orange peak in top panel of Fig. 4) has been noted previously (13). This region has the most densely packed SNPs between AKMV subclades. It spans approximately 6,200 nucleotides, encompassing a number of AKMV genes, spanning from the midpoint of AKMV-014 orthologs through AKMV-020. These genes encode important virulence factors, including an ankyrin, epidermal growth factor (EGF)-like growth factor, interleukin 1 (IL-1) receptor antagonist, ubiquitin ligase, and IL-18 binding proteins (Table S1). At this region, the nucleotide identity between the 2 AKMV subclades is ∼86%, significantly reduced from a genome-wide average of 99% identity. For comparison, this region contains greater genetic divergence between AKMV isolates than between many orthopoxvirus species (18). A BLASTn search identified CPXV clade B isolates as the most similar sequences in GenBank, with 92% identity. Genomic comparisons (via the Find Differences tool log-file) identified 1,694 SNPs between the AKMV subclades, 51% of which occur within this ∼6,200-nucleotide stretch (2.8% of genome) rather than dispersed across the genome. The mutation accumulation rate discrepancy between this region and the remainder of the genome, along with the higher similarity of this region to CPXV isolates, suggests a recombination event. Note that all isolates of the AKMV-VANI10/A39/A40 subclade contain the same single nucleotide deletion that fragmented the orthologs of the AKMV-014 gene (major histocompatibility complex class I [MHC1]-like protein), as well as the same internal 736-nt deletion within the AKMV-015 gene (ankyrin). BLASTn shows this ankyrin (with internal deletion) is unique, but the effect is unknown since numerous poxviruses have ankyrin fragments. Altogether, the results suggest the recombination event occurred after the divergence of the two AKMV subclades but prior to the divergence of the most recent common ancestor of the subclade into members AKMV-VANI10/A39/A40 (see orange arrow in Fig. 3). This is interesting because recombination and deletions (that lead to the loss of a functional gene) have been suggested to contribute to important evolutionary events such as the speciation and host range restrictions of variola virus (VARV) and camelpox virus (CMLV)/taterapox virus (TATV) (19).

**FIG 5:**
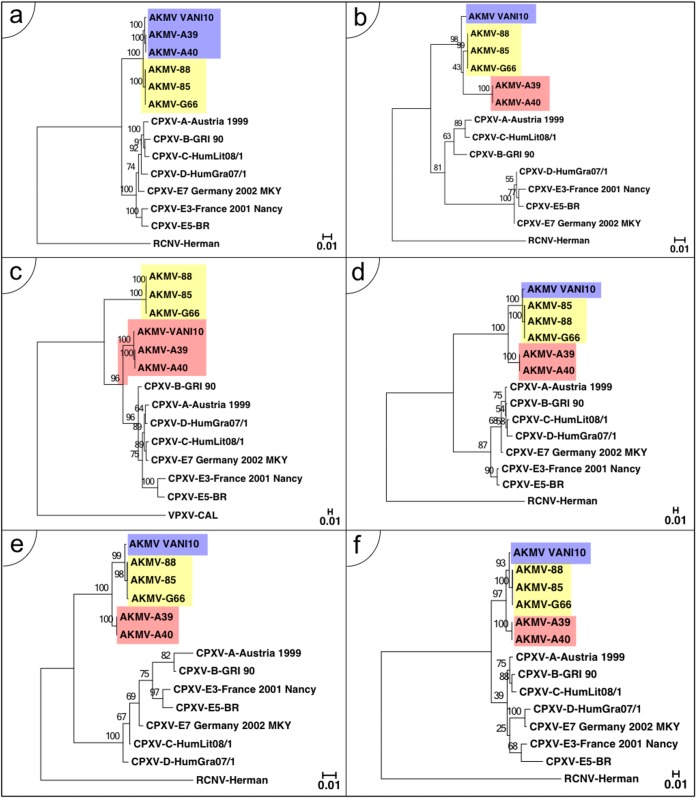
Maximum likelihood phylogenies of recombined regions. For each of the recombined regions identified by Base-By-Base in Fig. 4, nucleotide sequence alignments were extracted against a common set of CPXVs with orthologous regions and inputted into RAxML. The AKMV branching pattern in control core region (a) is compared to the recombinations identified at coding sequences of AKMV-009 (b), 014-020 (c), 175-177 (d), 199 (e), and 208 (f). Highlighted accordingly are the control AKMV-VANI10 subclade position (blue), AKMV-88 subclade position (yellow), and the shifted positions due to recombinations (red). The CPXVs include a strain from each clade: A-Austria 1990, B-GRI-90, C-HumLit081/1, D-HumGra07/1, E3-France2001Nancy, E5-BR, and E7-Germany2002MKY. Trees are rooted with raccoonpox virus (RCNV) except in panel c, which is missing orthologous RCNV region and is rooted with volepox virus (VPXV) instead. Gaps have been taken out of the multiple sequence alignment for panel f for better CPXV resolutions.

The other 4 SNP clusters are much smaller in scale and therefore more difficult to detect. However, these were observed in a comparison of AKMV-VANI10 and the A40/A39 strains (purple bars in Fig. 4, bottom) using the Find Differences tool of BBB which allows the user to recognize small clusters of SNPs that do not match the overall phylogeny. The 4 identified regions correspond to sequences encoding C-type lectin domain-containing protein (AKMV-009), profilin-like protein, type I membrane glycoprotein, VACV-Cop-A43.5R ortholog (-175 to -177), kelch-like protein (-199), and ankyrin protein (-208). At each of these recombined regions, their phylogenetic tree supports the BBB result and shows an altered AKMV branching pattern (Fig. 5). Thus, we demonstrate that the Find Differences tool used in conjunction with the Visual Summary report is able to quickly identify incongruent regions, without the hassle of creating an individual alignment and phylogeny of each gene. Although the significance of these recombination events is hard to infer through bioinformatics alone, switching of regions that contribute to virulence/host range could easily create novel viruses with altered characteristics. Finally, the graph also displays a small peak near nucleotide position 98,320 when comparing the G66 isolate to the 88/85 isolates (green bars in Fig. 4, bottom). This did not reveal any evidence of recombination but was found to result from the insertion of 7 Ns (placeholder for unresolved repeat sequence) in the genome sequence and shows the sensitivity of the “human eye” approach. Recombination events between vaccinia viruses are believed to be common because of the long history this virus has of passage in animals and tissue culture (20–22). Although fewer have been recognized in the genomes of “wild viruses,” examples have been recognized in CPXVs, variola viruses, and centapoxviruses (15, 19, 23).

## DISCUSSION

Several zoonotic orthopoxviruses are thought to be maintained in the environment by small mammal reservoirs (24–28). Most studies assessing OPXV infections in small mammals are serological, relying on the presence of antibodies to indicate previous OPXV infection (4, 25, 26, 29–36). This is due to the difficulty of finding naturally occurring active infections in a relatively small number of animals. However, active infections permit detection of viral DNA and collection of viable virus. Here, we present the first isolation of Akhmeta virus from small-mammal samples. This virus was originally identified in human infections of cattle herders near Jijeti, Georgia, in 2013; although live virus and DNA have not yet been recovered from cow samples collected in Georgia, anti-OPXV IgG-positive cows were identified in the original outbreak investigation. More work is needed to assess the prevalence of Akhmeta virus infections in Georgian cattle and other domestic animals.

Mammalian taxonomy in the South Caucasus is complex, and mammal species from three major regions (Europe, Asia, and the Middle East) often overlap. To further complicate these issues, some of these species (e.g., *Apodemus flavicollis* and A. ponticus) are closely related and morphologically difficult to distinguish (37). There is uncertainty in the literature as to whether or not the oriental subspecies of *A. flavicollis* and *A. ponticus* are actually the same taxon (38), as reciprocal monophyly of these two taxa is not always identified in phylogenetic analyses (39) and geographic distributions are not immutable. For these reasons, combined with geographic localities of reference samples and morphological data, we identified A39 and A40 specimens as *A. flavicollis*.

Of all the small-mammal genera sampled during this study, only samples collected from *Apodemus* (*A. uralensis* and *A. flavicollis*) were positive for viral DNA and live virus. Although this supports the hypothesis that *Apodemus* rodents play a role in the circulation and maintenance of AKMV in the environment and suggests they could possibly be the reservoir taxa, the much smaller sample sizes of the other taxa do not rule out those genera. More data are needed from additional locations and time points to make such a conclusion. Several reports point to *Microtus* and *Apodemus* rodents as potential reservoirs of cowpox viruses in Europe (25, 31, 32, 40, 41). Despite the large number of *Apodemus* samples examined in those studies, this is the first known isolation of an OPXV from *Apodemus* rodents. OPXVs are known to be able to infect a wide variety of mammals (42–44); therefore, it is not surprising that live AKMV was detected in samples from 2 closely related species of this genus. Antibody detection by enzyme-linked immunosorbent assay (ELISA) could suggest that other animal taxa are involved in the natural cycle of OPXVs, but current assays would not be able to specifically identify previous infections with AKMV.

**FIG 6:**
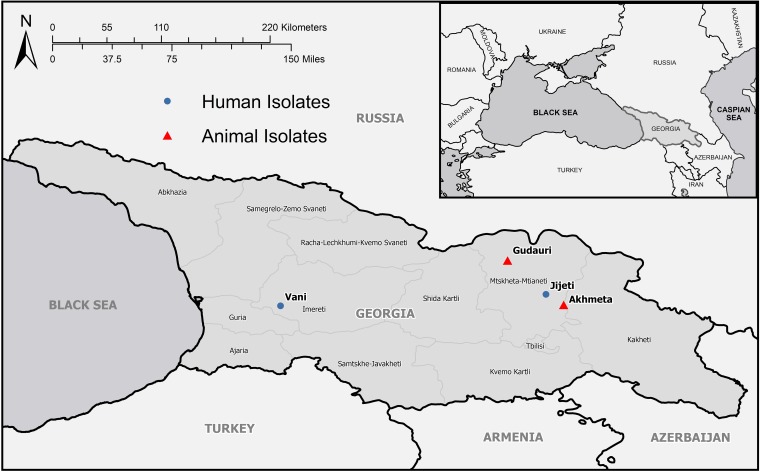
Map of where Akhmeta virus isolates were obtained from humans and animals in Georgia. Administrative boundaries were obtained from GADM (https://gadm.org); the map was created using ArcMap 10.5 (ESRI, Redlands, CA).

The AKMV genomes examined here separate into 2 distinct clades: AKMV-VANI10/A39/A40 and AKMV-13-85/13-88/G66. It appears that geographic distance does not explain the genetic variation observed in these genomes; the sampling location of Akhmeta (A39 and A40) is geographically closer to Gudauri (G66) and Jijeti (AKMV-13-85, 13-88) than to Vani (VANI10) (Fig. 6). The rodent hosts may also provide an additional explanation for this observed variation. Isolates obtained from A39 and A40 were derived from *A. flavicollis* rodents and G66 from *A. uralensis* rodents. This suggests the possibility that *A. flavicollis* may serve as host for the AKMV-VANI10/A39/A40 strain and *A. uralensis* may serve as host for AKMV-13-85/13-88/G66. However, it seems unlikely these associations would be exclusive, especially given the broad host range of OPXVs.

In this project, pox-like lesions, dried blood, and heart plus lung samples were the best samples used for molecular diagnostic screening. Of the 11 PCR-positive samples, 3 were lesion material, 3 were dried blood, 4 were pooled heart plus lung, and 1 was a pooled kidney plus spleen sample. It would be interesting to determine if there is a significant difference between detection in lung and heart tissues, since one might hypothesize that the lungs are initially infected via spread by aerosol or mutual grooming.

The AKMV-specific assay appeared to be more sensitive than the OPXV generic assay; threshold cycle (*C_t_*) values for each sample were lower with the AKMV assay than with the OPXV assay (Table 2). This assay could be used to screen potential AKMV samples from the field; however, it will only detect AKMV and it is likely that other OPXVs, including cowpox-like viruses, are also circulating in this region of the world. Given this information, future studies may want to use a combination of PCR assays for screening diagnostic specimens for OPXVs in Georgia. Interestingly, live virus was cultured from two animals with no apparent signs of pox-like lesions. This might reflect the testing of animals at different stages of infection (e.g., with lesion versus pre-/postlesion). However, multiple routes of infection could also explain these differences, e.g., aerosol/grooming to the lungs versus direct scratches to the skin. In all three animals with PCR-positive pox-like lesions, viable virus was recovered from the lesion material and, in one case, from heart plus lung tissue as well.

It is interesting that the AKMV genomes appear to have undergone several recombination events. Such genome changes may allow viral evolution to proceed more rapidly than the gradual accumulation of SNPs. Although it has been suggested that recombination at the virulence O1L gene (involved in replication) may have played a key role in the host switch/restriction and subsequent adaption of variola virus to humans (19), such events have likely contributed to the evolution of all poxviruses. In addition, the recognition of multiple zoonotic poxvirus infections of humans in recent years (1, 45, 46) suggests that there are probably many more poxviruses with the potential to infect humans circulating in wild animals, yet to be discovered. Since the infections of the wild animals appear to be generally mild and go unnoticed, humans may be the best sentinel animals, and increasing awareness of potential poxvirus infections should be encouraged.

## MATERIALS AND METHODS

Trapping locations were selected based on previous evidence of OPXV infections in cattle or humans, elevation, and habitat (Fig. 6). The Akhmeta field site (1,024-m elevation) is located near the original outbreak site that was investigated in 2013 and in an area where livestock are seasonally moved between the Kakheti and Mtskheta-Mtianeti regions. The Gudauri site has an elevation of 1,775 m, which is similar to the case patient’s property (1,647 m) from the original investigation of the 2013 outbreak. Both sites were generally forested; however, some trap lines at the Gudauri site were placed on the edge of farm and pastureland used as grazing areas for livestock.

As part of a larger longitudinal research effort, these collection trips were conducted in July (Gudauri) and October (Akhmeta) 2016. Sherman live traps baited with peanut butter and oats were placed in the evening and checked each morning for 3 consecutive nights. Captured animals were transported to a central processing area where they were anesthetized with isoflurane prior to data collection and euthanasia. Standard measurements were recorded to assist with species identification, and each animal was assessed for the presence of wounds and pox-like lesions. Following euthanasia, heart plus lung (pooled for each animal), spleen plus kidney (pooled for each animal), liver, and if present, lesion samples were collected and stored in a liquid nitrogen dry shipper while in the field. Dried blood samples were collected on Nobuto filter paper strips (Advantec, San Diego, CA). All work was approved by the CDC Institutional Animal Care and Use Committee (IACUC) under protocol 2703DOTMULX.

For DNA extraction and sample processing, the samples were homogenized in 500 μl phosphate-buffered saline (PBS; pH 7.4) with a sterile stainless-steel bead in a Geno grinder (SPEX SamplePrep, LLC, Metuchen, NJ). One hundred microliters of this homogenate was used for DNA extraction in a MagMAX Express-96 Deep Well magnetic particle processor (Thermo Fisher Scientific, Waltham, MA) with a MagMAX DNA Multi-Sample Ultra kit. The presence of viral DNA was assessed using real-time PCR to detect the E9L gene (DNA polymerase) of orthopoxviruses as previously described (47). A second Akhmeta-specific PCR was developed and conducted on OPXV-positive samples.

The AKMV-specific primer and probe sequences were selected based on genomic sequence alignments of the published Akhmeta viral genomes (GenBank numbers MH607141.1, MH607142.1, and MH607143.1) and other OPXVs. The probe used in this assay is highly specific for AKMV and targets a gene encoding a hypothetical protein of 354 amino acids (F11L of VACV-Copenhagen). The assay included the forward primer (5′-GAA CTC AAG GAT TTA CTC AAT GTT ACA), the reverse primer (5′-TCT GCG GGT CT A AAA TTT CCA TCT), and the probe (5′-6-carboxyfluorescein [FAM]-AAC CGG TCA CCA TCC CCG ACA TCA AG-black hole quencher 1 [BHQ1]). The detection probe contains a 5′ reporter molecule (FAM) (Glen Research, Sterling, VA) and a 3′ quencher molecule (BHQ1) (Molecular Probes, Eugene, OR). PCR protocol conditions were optimized according to standard protocols (protocol 0430449; Applied Biosystems, Foster City, CA) by adjusting primer and probe concentrations and thermal cycling conditions. Each 20-μl reaction mixture contained 0.5 μl of each primer (20 μM), 0.5 μl of probe (10 μM), 10 μl of TaqMan Advanced 2× buffer (Applied Biosystems, Foster City, CA), 3.5 μl of nuclease-free water, and 5 μl of DNA template (sample DNA). Thermal cycling conditions for the ABI 7500 Fast (Applied Biosystems, Foster City, CA) were one cycle of 95°C for 10 min followed by 40 cycles of 95°C for 3 s and 60°C for 30 s. PCR ampliﬁcation is based on ﬂuorescence emission after annealing/elongation (60°C).

Samples positive for AKMV DNA were added to cell culture for virus isolation and viral titration. Homogenized samples from the DNA extraction process were added to BSC-40 cell monolayers (African green monkey kidney cell line) in 6-well plates (titration) or T-25 cell culture flasks (viral isolation) and incubated at 37°C with 5% CO_2_. Titrations were conducted using 10-fold dilutions of tissue homogenates, and following a 72-h incubation, the samples were fixed/stained with formalin/crystal violet to reveal plaques (48).

For each of the viral isolates, DNA was extracted from infected cell culture and sequencing was performed using the Illumina HiSeq 2500 platform (Otogenetics, Norcross, GA, USA). Paired-end reads were used to assemble the genomes in CLC Genomics Workbench 11.0 (Qiagen, Aarhus, Denmark) according to a previously published methodology (13). The genomes were annotated with GATU (49) using AKMV-88 as a reference. Percentage of amino acid identity was obtained by BLASTp (50) for each gene. Using the BBB editor (17), genomes were aligned with MAFFT and compared to the previously described AKMV isolates obtained from human cases (AKMV-88, AKMV-85, and VANI10; (13). The core regions of the 3 new AKMV genomes (orthologous to VACV-Cop-F9L to A24R) were aligned with 24 OPXV representatives (listed in Fig. 3A). Using this core nucleotide alignment, a maximum likelihood phylogenetic analysis with 1,000 bootstrap tests was conducted in RAxML v 8.2.10 (51) under the GTRGAMMA model to reconstruct the relationship between these viruses. Another tree was created by GToTree (52), which recognized and concatenated amino acid sequences of protein domains from conserved chordopoxvirus genes through a customized poxvirus HMM. The BBB built-in reports Find Differences and Visual Summary were used to examine genome-wide recombination events. Find Differences logs were exported and plotted in Excel to combine different analyses. The Visual Summary report allowed for interactive zooming and scanning and visualization of the SNP and indel distributions between genomes.

Viral isolates, their associated genomes, and the individual animals were named using the first letter of the field location (A, Akhmeta; G, Gudauri) and a sequential accession number assigned during field processing. All animals were identified to the genus or species level in the field; however, to confirm these identifications, a 400-bp fragment of the mitochondrial cytochrome *b* (*CytB*) gene was amplified from the animals with AKMV-positive samples using primers MVZ05 and R400 (53, 54) (CDC Core Facility). The purified PCR products were then sequenced on an ABI PRISM 3130-Avant genetic analyzer (Applied Biosystems, Foster City, CA, USA); sequences were assembled and proofed with Geneious v10.2.2 software (Biomatters Ltd., Auckland, New Zealand). Representative sequences of the *Apodemus* species known to occur in the region were selected for Bayesian inference analysis using MrBayes v3.2.6 (55, 56) to confirm the megaBLAST species identification of these specimens.

### Data availability.

Cytochrome *b* sequences were deposited in GenBank (MK938304 to MK938308). Full genome sequences for G66, A39, and A40 were deposited in GenBank under accession numbers MN244296, MN244297, and MN244298, respectively.

## Supplementary Material

Supplemental file 1
