# Supplementary material for: Isolation and Characterization of Akhmeta Virus from Wild-Caught Rodents (Apodemus spp.) in Georgia
Source: J Virol. 2019 Nov 26;93(24):e00966-19. doi: 10.1128/JVI.00966-19 (PMC6880181; doi:10.1128/JVI.00966-19)
Supplement: Supplemental file 1 [file JVI.00966-19-s0001.pdf]

Supplemental Table S1. AKMV comparison table gene by gene (against reference strain AKMV-88)

Percentage of amino acid identity (% id) and protein size differences (% size) that are outside of the average standard deviation of the genes for each genome are highlighted in red and yellow, respectively; truncation and extension of genes are dictated by % size when compared to the reference

	AKMV-88							85	G66	VANI	A40	A39	85	G66	VANI	A40	A39
	ORF	Function	CPXV-BR	CPXV-GRI-90	VACV-Cop	Position	AA size	% id					% size				
ITR region	AKMV001	Chemokine binding protein	3	D1L	C23L	2675-1923	250	100	100	98.8	98.4	98.4	100	100	100	100	100
	AKMV002	TNF receptor (CrmB)	5	D2L	C22L	3840-2794	348	100	100	99.4	99.7	99.7	100	100	100	100	100
	AKMV003	Ankyrin	6	D3L	C19L	5696-3927	589	100	100	99.3	99.3	99.3	100	100	100	100	100
	AKMV004	-	7	-	-	5842-5708	44	100	100	100	100	100	100	100	84.1	84.1	84.1
	AKMV005	Ankyrin	8	D4L	C17L	7976-5961	671	100	100	98.1	98.8	98.8	100	100	100	100	100
	AKMV006	-	9	D5L	C16L	8622-8161	153	100	100	98	98.7	98.7	100	100	100	100	100
Left terminal region	AKMV007	BTB Kelch-domain containing protein	10	D7L	A55R	10036-9215	273	100	100	98.5	98.5	98.5	100	100	100	100	100
	AKMV008	Ankyrin	11	D8L	B20R	12098-10119	659	100	100	98.8	98.6	98.6	100	33.4	99.7	100	100
	AKMV009	C-type lectin domain containing protein	12	D10L	-	12610-12131	159	100	100	96.9	93.7	93.7	100	100	100	100	100
	AKMV010	TNF receptor (CrmB)	14	D12L	C22L	13360-12752	202	100	100	98.5	100	100	100	100	99.5	102	102
	AKMV011	TNF-alpha receptor-like protein	15	D13L	-	13692-13357	111	100	100	100	99.1	99.1	100	100	100	101	101
	AKMV012	Ankyrin	16	D14L	B18R	16100-13800	766	100	99.9	99.2	99.5	99.5	100	100	63.3	100	100
	AKMV013	Ankyrin	17	C1L	-	17807-16497	436	100	100	99.5	99.1	99.1	100	100	100	100	100
	AKMV014	MHC1-like protein fragment (MPXV-Z-N3R)	18	C2L	-	18439-17906	177	100	100	96.5	95.6	95.6	100	100	64.4	64.4	64.4
	AKMV015	Ankyrin	19	C3L	B18R	20952-18547	801	100	99.4	74.7	74.5	74.5	100	99.6	69.4	69.4	69.4
	AKMV016	Host range protein	20	C4L	-	21566-21060	168	100	100	74.3	72.8	72.8	100	100	102	101	101
	AKMV017	Secreted EGF-like protein	21	C5R	C11R	21741-22154	137	100	100	76.3	75.5	75.5	100	100	101	101	101
	AKMV018	IL-1 receptor antagonist	22	C6L	C10L	23288-22290	332	100	100	84.7	84.4	84.4	100	100	99.4	99.4	99.4
	AKMV019	Ubiquitin ligase	23	C7R	-	23830-24573	247	100	100	85.1	85.5	85.5	100	100	98	98	98
	AKMV020	Soluble IL-18 binding protein	24	C8L	-	25144-24743	133	100	100	91.1	91.1	91.1	100	100	101	101	101
	AKMV021	Ankyrin	25	C9L	-	27193-25175	672	100	99.9	97.6	97.5	97.5	100	100	100	100	100
	AKMV022	Ankyrin	26	C10L	-	27538-27347	63	100	100	100	100	100	100	100	100	100	100
	AKMV023	Type I IFN resistance	27	C11L	C9L	29652-27718	644	100	100	97.6	95.6	95.6	100	100	101	103	103
	AKMV024	-	28	C12L	C8L	30247-29693	184	100	100	98.9	98.9	98.9	100	100	100	100	100
	AKMV025	Type 1 IFN inhibitor	29	C13L	C7L	30772-30320	150	100	100	100	100	100	100	100	100	100	100
	AKMV026	Bcl-2-like protein, IFN-beta inhibitor	30	C14L	C6L	31481-30990	163	100	100	95.8	98.1	98.1	100	100	103	104	104
	AKMV027	Kelch-like protein	31	-	C5L	31781-31641	46	100	100	100	100	100	100	100	43.5	73.9	73.9
	AKMV028	Kelch-like protein	32	C15L	C5L	32251-31799	150	100	100	98.8	96.6	96.6	100	100	68	127	127
	AKMV029	IL-1 receptor antagonist	33	C16L	C10L	33269-32328	313	100	99.7	97.4	98.7	98.7	100	100	100	100	100
	AKMV030	Complement binding (secreted)	34	C17L	C3L	34128-33337	263	100	100	97.3	98.1	98.1	100	100	100	100	100
	AKMV031	POZ/BTB Kelch domain protein	35	C18L	C2L	35723-34185	512	100	100	99	99.4	99.4	100	100	100	100	100
	AKMV032	Putative TLR signalling inhibitor	36	C19L	C1L	36479-35790	229	100	100	99.1	98.3	98.3	100	100	92.1	100	100
	AKMV033	NF-κB inhibitor	37	Q1L	N1L	36819-36466	117	100	100	99.1	99.1	99.1	100	100	100	100	100
	AKMV034	Alpha amanitin-sensitive protein	38	Q2L	N2L	37481-36948	177	100	100	99.4	99.4	99.4	100	100	100	100	100
	AKMV035	Ankyrin	39	P1L	M1L	38863-37523	446	100	100	99.6	99.6	99.6	100	100	100	100	100
	AKMV036	NFkB inhibitor	40	P2L	M2L	39570-38908	220	100	100	100	99.5	99.5	100	100	100	100	100
	AKMV037	Ankyrin	41	M1L	K1L	40556-39702	284	100	100	99.6	99.3	99.3	100	100	100	100	100
	AKMV038	Serpin	42	M2L	K2L	41867-40746	373	100	100	99.7	99.7	99.7	100	100	100	100	100
	AKMV039	IFN resistance, PKR/eIF-alpha inhibitor	43	M3L	K3L	42183-41917	88	100	100	100	100	100	100	100	100	100	100
	AKMV040	Phospholipase-D-like protein	44	M4L	K4L	43518-42244	424	100	100	99.5	99.5	99.5	100	100	100	100	100
	AKMV041	Monoglyceride lipase	45	M5L	K5L	44378-43545	277	100	100	99.6	100	100	100	100	100	100	100
	AKMV042	Host immune response repressor	46	M6R	K7R	44518-44967	149	100	100	98.7	98.7	98.7	100	100	100	100	100
	AKMV043	-	47	-	-	44997-45164	55	100	100	100	98.2	98.2	100	100	100	100	100
	AKMV044	Caspase-9 (apoptosis) inhibitor (mitochondrial-associated)	48	G1L	F1L	45772-45038	244	100	95.1	99.2	98	98	100	95.1	99.2	100	100
	AKMV045	dUTPase	49	G2L	F2L	46206-45772	144	100	100	99.3	99.3	99.3	100	100	100	100	100
	AKMV046	Kelch-like protein	50	G3L	F3L	47682-46240	480	100	100	99.8	99.8	99.8	100	100	100	100	100
	AKMV047	Ribonucleotide reductase small subunit	51	G4L	F4L	48652-47693	319	100	100	99.7	100	100	100	100	100	100	100
	AKMV048	36kDa major membrane protein	52	G5L	F5L	49653-48685	322	100	100	99.1	98.8	98.8	100	100	100	100	100
	AKMV049	-	53	G6L	F6L	49904-49683	73	100	100	97.3	97.3	97.3	100	100	101	101	101
	AKMV050	-	54	G7L	F7L	50171-49920	83	100	100	95.4	95.4	95.4	100	100	105	105	105
	AKMV051	Cytoplasmic protein	55	G8L	F8L	50517-50320	65	100	100	100	100	100	100	100	100	100	100
	AKMV052	S-S bond formation pathway protein substrate	56	G9L	F9L	51215-50577	212	100	100	100	99.5	99.5	100	100	100	100	100
	AKMV053	Essential Ser/Thr kinase morph	57	G10L	F10L	52521-51202	439	100	100	99.5	99.8	99.8	100	100	100	100	100
	AKMV054	Overlapped with a bigger ORF															
	AKMV055	RhoA signalling inhibitor, virus release protein	59	G11L	F11L	53608-52544	354	100	100	99.2	99.7	99.7	100	100	100	100	100
	AKMV056	EEV maturation protein	60	G12L	F12L	55577-53667	636	100	100	99.1	99.4	99.4	100	100	100	100	100
	AKMV057	Palymytilated EEV membrane glycoprotein	61	G13L	F13L	56728-55610	372	100	100	100	100	100	100	100	100	100	100
	AKMV058	-	62	G14L	F14L	56973-56746	75	100	100	100	100	100	100	100	100	100	100
	AKMV059	IMV protein	-	-	F14.5L	57173-57024	49	100	100	100	100	100	100	100	100	100	100
	AKMV060	-	64	G15L	F15L	57721-57245	158	100	100	100	100	100	100	100	100	100	100
	AKMV061	Non-functional serine recombinase	65	G16L	F16L	58423-57728	231	100	100	100	99.6	99.6	100	100	100	100	100
	AKMV062	DNA-binding phosphoprotein (VP11)	66	G17R	F17R	58486-58791	101	100	100	100	100	100	100	100	100	100	100
	AKMV063	Poly(A) polymerase catalytic subunit (VP55)	67	F1L	E1L	60227-58788	479	100	100	100	100	100	100	100	100	100	100
	AKMV064	IEV morphogenesis	68	F2L	E2L	62437-60224	737	100	100	99.3	99.5	99.5	100	100	100	100	100
	AKMV065	dsRNA-binding protein, IFN resistance/PKR inhibitor (Z-DNA binding)	69	F3L	E3L	63120-62548	190	100	100	100	100	100	100	100	100	100	100
	AKMV066	RNA polymerase (RPO30) subunit	70	F4L	E4L	63956-63177	259	100	100	99.2	99.2	99.2	100	100	100	100	100
	AKMV067	Virosome component	71	F5R	E5R	64005-65039	344	100	100	99.1	99.7	99.7	100	100	100	100	100
	AKMV068	Virion protein	72	F6R	E6R	65251-66954	567	100	100	99.6	99.6	99.6	100	100	99.8	100	100
	AKMV069	Myristylated protein	73	F7R	E7R	67046-67549	167	100	100	99.4	99.4	99.4	100	100	100	100	100
	AKMV070	ER-localized membrane protein, virion core protein	74	F8R	E8R	67654-68475	273	100	100	99.6	99.3	99.3	100	100	100	100	100
	AKMV071	DNA polymerase	75	F9L	E9L	71513-68481	1010	100	100	99.7	99.8	99.8	100	100	100	100	100
	AKMV072	Sulfhydryl oxidase (FAD-linked)	76	F10R	E10R	71545-71835	96	100	100	100	99	99	100	100	100	100	100
	AKMV073	Virion core protein	77	F11L	E11L	72219-71830	129	100	100	97.7	98.5	98.5	100	100	100	100	100
	AKMV074	Membrane protein	78	R1L	O1L	74206-72206	666	100	100	99.9	99.9	99.9	100	100	100	100	100

	AKMV075	Overlapped with a bigger ORF															
	AKMV076	Glutaredoxin 1	79	R2L	O2L	74580-74254	108	100	100	99.1	100	100	100	100	100	100	100
	AKMV077	DNA-binding core protein	80	L1L	I1L	75664-74726	312	100	100	100	100	100	100	100	100	100	100
	AKMV078	IMV membrane protein	81	L2L	I2L	75895-75671	74	100	100	100	98.6	98.6	100	100	100	100	100
	AKMV079	ssDNA-binding phosphoprotein	82	L3L	I3L	76705-75896	269	100	100	100	100	100	100	100	100	100	100
	AKMV080	Ribonucleotide reductase large subunit	83	L4L	I4L	79105-76790	771	100	100	99.4	99.2	99.2	100	100	100	100	100
	AKMV081	IMV protein VP13	84	L5L	I5L	79372-79133	79	100	100	100	100	100	100	100	100	100	100
	AKMV082	Telomere-binding protein	85	L6L	I6L	80539-79391	382	100	100	99.5	99.2	99.2	100	100	100	100	100
	AKMV083	Virion core cysteine protease	86	L7L	I7L	81803-80532	423	100	100	99.8	99.8	99.8	100	100	100	100	100
	AKMV084	RNA helicase, DExH-NPH-II domain	87	L8R	I8R	81809-83842	677	99.9	100	98.7	98.7	98.7	100	100	100	100	100
	AKMV085	Metalloprotease	88	H1L	G1L	85621-83846	591	100	100	99.8	100	100	100	100	100	100	100
	AKMV086	Entry/fusion complex component	89	H2L	G3L	85953-85618	111	100	100	99.1	99.1	99.1	100	100	100	100	100
	AKMV087	VLTF (late transcription elongation factor)	90	H3R	G2R	85947-86609	220	100	100	98.6	100	100	100	100	100	100	100
	AKMV088	Glutaredoxin-like protein	91	H4L	G4L	86953-86579	124	100	100	100	100	100	100	100	100	100	100
	AKMV089	FEN1-like nuclease	92	H5R	G5R	86956-88254	432	100	100	99.3	99.1	99.1	100	100	100	100	100
	AKMV090	RNA polymerase (RPO7) subunit	93	H6R	G5.5R	88262-88453	63	100	100	100	98.4	98.4	100	100	100	100	100
	AKMV091	NLPc/P60 superfamily protein	94	H7R	G6R	88455-88931	158	100	100	98.7	98.7	98.7	100	100	100	100	100
	AKMV092	Virion phosphoprotein, early morphogenesis	95	H8L	G7L	90032-88917	371	99.7	100	98.7	99.7	99.7	100	100	100	100	100
	AKMV093	Overlapped with a bigger ORF															
	AKMV094	VLTF-1 (late transcription factor 1)	97	H9R	G8R	90063-90845	260	100	100	100	100	100	100	100	100	100	100
	AKMV095	Entry/fusion complex component, myristylprotein	98	H10R	G9R	90873-91895	340	100	100	99.1	99.1	99.1	100	100	100	100	100
	AKMV096	IMV membrane protein	99	N1R	L1R	91896-92648	250	100	100	100	100	100	100	100	100	100	100
	AKMV097	Crescent membrane and immature virion formation protein	100	N2R	L2R	92680-92943	87	100	100	96.6	96.6	96.6	100	100	100	100	100
	AKMV098	Internal virion protein	101	N3L	L3L	93994-92933	353	100	99.7	99.4	99.7	99.7	100	100	100	100	100
	AKMV099	ss/dsDNA binding protein (VP8)	102	N4R	L4R	94019-94774	251	100	100	100	100	100	100	100	100	100	100
	AKMV100	Entry and fusion IMV protein	103	N5R	L5R	94784-95170	128	100	100	100	100	100	100	100	100	100	100
	AKMV101	Virion morph	104	S1R	J1R	95154-95588	144	100	100	100	100	100	100	100	100	100	100
	AKMV102	Thymidine kinase	105	S2R	J2R	95604-96137	177	100	100	100	99.4	99.4	100	100	100	100	100
**Core**	AKMV103	Poly (A) polymerase small subunit (VP39)	106	O1R	J3R	96204-97205	333	100	100	99.7	100	100	100	100	100	100	100
	AKMV104	RNA polymerase (RPO22) subunit	107	O2R	J4R	97120-97677	185	100	100	99.5	99.5	99.5	100	100	100	100	100
	AKMV105	IMV membrane protein	108	O3L	J5L	98254-97853	133	100	100	100	100	100	100	100	100	100	100
	AKMV106	RNA polymerase (RPO147) subunit	109	O4R	J6R	98360-102220	1286	100	100	100	99.8	99.8	100	100	100	100	100
	AKMV107	Tyr/Ser phosphatase, IFN-gamma inhibitor	110	J1L	H1L	102732-102217	171	100	100	100	100	100	100	100	100	100	100
	AKMV108	IMV membrane protein	111	J2R	H2R	102746-103315	189	100	100	100	100	100	100	100	100	100	100
	AKMV109	IMV heparin binding surface protein (p35)	112	J3L	H3L	104292-103318	324	100	100	99.7	99.4	99.4	100	100	100	100	100
	AKMV110	RAP94 (RNA polymerase-associated protein)	113	J4L	H4L	106680-104293	795	100	100	99.5	99.6	99.6	100	100	100	100	100
	AKMV111	VLTF-4 (late transcription factor 4)	114	J5R	H5R	106866-107492	208	100	100	99	98.6	98.6	100	100	100	100	100
	AKMV112	DNA topoisomerase type I	115	J6R	H6R	107493-108437	314	100	100	99	99.7	99.7	100	100	100	100	100
	AKMV113	-	116	-	-	108593-108393	66	100	100	98.5	97	97	100	100	100	100	100
	AKMV114	Crescent membrane and immature virion formation	117	J7R	H7R	108475-108915	146	100	100	100	100	100	100	100	100	100	100
	AKMV115	mRNA capping enzyme large subunit	118	E1R	D1R	108959-111487	842	100	100	99.9	100	100	100	100	100	100	100
	AKMV116	Virion core protein	119	E2L	D2L	111886-111446	146	100	100	100	100	100	100	100	100	100	100
	AKMV117	Virion core protein	120	E3R	D3R	111879-112592	237	100	100	99.6	99.6	99.6	100	100	100	100	100
	AKMV118	Uracil-DNA glycosylase, DNA polymerase processivity factor	121	E4R	D4R	112592-113248	218	100	100	99.1	99.1	99.1	100	100	100	100	100
	AKMV119	NTPase, DNA primase	122	E5R	D5R	113280-115637	785	100	100	99.7	99.9	99.9	100	100	100	100	100
	AKMV120	Morphogenesis, VETF-s (early transcription factor small subunit)	123	E6R	D6R	115678-117591	637	100	100	99.8	99.7	99.7	100	100	100	100	100
	AKMV121	RNA polymerase (RPO18) subunit	124	E7R	D7R	117618-118103	161	100	100	100	100	100	100	100	100	100	100
	AKMV122	Carbonic anhydrase, GAG-binding IMV membrane protein	125	E8L	D8L	118980-118066	304	100	100	99	99.3	99.3	100	100	100	100	100
	AKMV123	mRNA decapping enzyme	126	E9R	D9R	119022-119663	213	100	100	99.1	99.1	99.1	100	100	100	100	100
	AKMV124	mRNA decapping enzyme	127	E10R	D10R	119660-120406	248	100	100	98.8	98.8	98.8	100	100	100	100	100
	AKMV125	ATPase, NPH1	128	E11L	D11L	122302-120407	631	100	100	99.5	99.5	99.5	100	100	100	100	100
	AKMV126	mRNA capping enzyme small subunit	129	E12L	D12L	123199-122336	287	100	100	99.7	99.3	99.3	100	100	100	100	100
	AKMV127	Overlapped with a bigger ORF															
	AKMV128	Trimeric virion coat protein (rifampicin resistance)	131	E13L	D13L	124885-123230	551	100	99.8	99.6	99.6	99.6	100	100	100	100	100
	AKMV129	VLTF-2 (late transcription factor 2)	132	A1L	A1L	125361-124909	150	100	100	99.3	99.3	99.3	100	100	100	100	100
	AKMV130	VLTF-3 (late transcription factor 3)	133	A2L	A2L	126056-125382	224	100	100	100	100	100	100	100	100	100	100
	AKMV131	S-S bond formation pathway protein	134	A3L	A2.5L	126283-126053	76	100	100	98.7	98.7	98.7	100	100	100	100	100
	AKMV132	P4b precursor	135	A4L	A3L	128232-126298	644	100	100	99.7	99.7	99.7	100	100	100	100	100
	AKMV133	39kDa virion core protein	136	A5L	A4L	129133-128285	282	100	99.6	98.9	98.2	98.2	100	99.6	99.6	99.6	99.6
	AKMV134	RNA polymerase (RPO19) subunit	137	A6R	A5R	129171-129665	164	100	100	100	100	100	100	100	100	100	100
	AKMV135	Viral membrane assembly proteins (VMAP), core protein	138	A7L	A6L	130780-129662	372	100	100	100	100	100	100	100	100	100	100
	AKMV136	VETF-L (early transcription factor large subunit)	139	A8L	A7L	132936-130804	710	100	100	99.7	99.7	99.7	100	100	100	100	100
	AKMV137	VITF-3-s (intermediate transcription factor small subunit)	140	A9R	A8R	132990-133856	288	100	100	100	100	100	100	100	100	100	100
	AKMV138	Viral membrane associated, early morphogenesis protein	141	A10L	A9L	134195-133893	100	100	100	96.2	98	98	100	108	114	114	114
	AKMV139	P4a precursor	142	A11L	A10L	136874-134196	892	100	100	99.9	99.9	99.9	100	100	100	100	100
	AKMV140	Viral membrane formation	143	A12R	A11R	136889-137845	318	100	100	100	100	100	100	100	100	100	100
	AKMV141	Virion core and cleavage processing protein	144	A13L	A12L	138419-137847	190	100	100	100	100	100	100	100	100	100	100
	AKMV142	IMV membrane protein, virion maturation	145	A14L	A13L	138661-138443	72	100	100	98.6	95.8	95.8	100	100	100	97.2	97.2
	AKMV143	Essential IMV membrane protein	146	A15L	A14L	139040-138768	90	100	100	100	100	100	100	100	100	100	100
	AKMV144	Non-essential IMV membrane protein	147	-	A14.5L	139218-139057	53	100	100	100	100	100	100	100	100	100	100
	AKMV145	Core protein	148	A16L	A15L	139492-139208	94	100	100	100	100	100	100	100	100	100	100
	AKMV146	Myristylated protein, essential for entry/fusion	149	A17L	A16L	140609-139476	377	100	100	100	99.7	99.7	100	100	100	100	100
	AKMV147	IMV membrane protein	150	A18L	A17L	141235-140612	207	100	100	99.5	98.6	98.6	100	100	100	100	100
	AKMV148	DNA helicase, transcript release factor	151	A19R	A18R	141250-142734	494	100	100	99.8	99.8	99.8	100	100	100	100	100
	AKMV149	Zinc finger-like protein	152	A20L	A19L	142948-142715	77	100	100	98.7	97.4	97.4	100	100	100	100	100
	AKMV150	Overlapped with a bigger ORF															
	AKMV151	IMV membrane protein, entry/fusion complex component	153	A21L	A21L	143302-142949	117	100	100	100	100	100	100	100	100	100	100
	AKMV152	DNA polymerase processivity factor	154	A22R	A20R	143301-144581	426	100	100	99.8	99.5	99.5	100	100	100	100	100
	AKMV153	Holliday junction resolvase	155	A23R	A22R	144511-145074	187	100	100	100	100	100	100	100	100	100	100
	AKMV154	VITF-3-L (intermediate transcription factor large subunit)	156	A24R	A23R	145094-146242	382	100	100	99.7	99.5	99.5	100	100	100	100	100
	AKMV155	RNA polymerase (RPO132) subunit	157	A25R	A24R	146239-149733	1164	100	100	99.8	99.9	99.9	100	100	100	100	100
	AKMV156	A type inclusion protein	158	A26L	A25L	153367-149726	1213	100	100	98.9	98.1	98.1	100	100	99.5	98.7	98.7
	AKMV157	P4c precursor	159	A27L	A26L	154972-153413	519	100	99	98.7	98.3	98.3	100	101	100	101	101

Right terminal region	AKMV158	IMV surface protein, fusion protein	162	A28L	A27L	155356-155024	110	100	100	100	100	100	100	100	100	100	100
	AKMV159	IMV MP/Virus entry	163	A29L	A28L	155797-155357	146	100	100	97.9	98.6	98.6	100	100	100	100	100
	AKMV160	RNA polymerase (RPO35) subunit	164	A30L	A29L	156715-155798	305	100	100	100	100	100	100	100	100	100	100
	AKMV161	IMV protein	165	A31L	A30L	156911-156678	77	100	100	98.7	100	100	100	100	100	100	100
	AKMV162	-	166	A32R	A31R	157071-157454	127	100	100	100	100	100	100	100	100	100	100
	AKMV163	ATPase/DNA packaging protein	167	A33L	A32L	158323-157424	299	100	100	99.7	100	100	100	100	100	100	100
	AKMV164	EEV membrane phosphoglycoprotein, C-type lectin-like domain	168	A34R	A33R	158363-158920	185	100	100	98.9	98.9	98.9	100	100	100	100	100
	AKMV165	C-type lectin-like IEV/EEV glycoprotein	169	A35R	A34R	158944-159450	168	100	100	100	100	100	100	100	100	100	100
	AKMV166	Overlapped with a bigger ORF															
	AKMV167	MHC class II antigen presentation inhibitor	171	A36R	A35R	159495-160025	176	100	100	99.4	99.4	99.4	100	100	100	100	100
	AKMV168	IEV transmembrane phosphoprotein	172	A37R	A36R	160092-160760	222	99.1	100	98.6	99.1	99.1	99.1	100	100	100	100
	AKMV169	-	173	A38R	A37R	160825-161616	263	100	100	100	100	100	100	100	100	100	100
	AKMV170	-	174	A39R	-	161725-161913	62	100	100	98.4	100	100	100	100	100	100	100
	AKMV171	CD47-like, integral membrane protein	175	A40L	A38L	162743-161910	277	100	100	99.6	99.6	99.6	100	100	100	100	100
	AKMV172	Semaphorin	176	A41R	A39R	162759-163955	398	99.7	100	99.2	98.2	98.2	100	100	100	100	100
	AKMV173	Lectin homolog	177	A42R	A40R	163982-164503	173	100	100	97.1	96.6	96.6	100	100	100	101	101
	AKMV174	Chemokine binding protein	178	A43L	A41L	165280-164618	220	100	100	99.1	98.6	98.6	100	100	100	100	100
	AKMV175	Profilin-like protein, ATI-localized	179	A44R	A42R	165462-165863	133	100	100	100	90.2	90.2	100	100	100	100	100
	AKMV176	Type I membrane glycoprotein	180	A45R	A43R	165904-166515	203	100	100	98.5	94.1	94.1	100	100	100	100	100
	AKMV177	-	181	A46R	A43.5R	166534-166761	75	100	100	94.7	92	92	100	100	100	100	100
	AKMV178	3β-hydroxysteroid dehydrogenase/δ 5->4 isomerase	182	A47L	A44L	167920-166886	344	100	99.7	99.7	97.7	97.7	100	100	100	100	100
	AKMV179	Inactive Cu-Zn superoxide dismutase-like virion protein	183	A48R	A45R	167968-168345	125	100	100	100	100	100	100	100	100	100	100
	AKMV180	Toll/IL-1 receptor-like protein; signaling inhibitor	184	A49R	A46R	168335-169057	240	100	100	99.2	99.2	99.2	100	100	100	100	100
	AKMV181	Immunoprevalent protein	185	A50L	A47L	169892-169158	244	100	100	100	100	100	100	100	100	100	100
	AKMV182	Thymidylate kinase	186	A51R	A48R	169991-170605	204	100	100	100	100	100	100	30.4	100	100	100
	AKMV183	Putative phosphotransferase/anion transport protein	187	A52R	A49R	170654-171142	162	100	100	97.5	98.1	98.1	100	100	100	100	100
	AKMV184	ATP-dependent DNA ligase	188	A53R	A50R	171174-172838	554	100	100	100	99.8	99.8	100	100	100	100	100
	AKMV185	-	189	A54R	A51R	172905-173909	334	100	100	100	99.7	99.7	100	100	100	100	100
	AKMV186	Toll/IL-1 receptor-like protein; IL-1, NFkB signalling inhibitor	190	A55R	A52R	173979-174563	194	100	100	100	100	100	100	100	100	100	100
	AKMV187	TNF receptor (CrmC)	191	A56R	A53R	174913-175467	184	100	100	97.8	98.9	98.9	100	100	100	100	100
	AKMV188	BTB Kelch-domain containing protein	193	A57R	A55R	175708-177402	564	100	100	99.8	99.8	99.8	100	100	100	100	100
	AKMV189	Hemagglutinin	194	A58R	A56R	177452-178393	313	100	100	99.4	99	99	100	100	100	100	100
	AKMV190	Guanylate kinase	195	A59R	A56.5R	178410-179003	197	100	100	99.5	100	100	100	100	100	100	100
	AKMV191	Ser/Thr Kinase	196	B1R	B1R	179144-180046	300	100	100	100	100	100	100	100	99.7	99.7	99.7
	AKMV192	Schlafen	197	B2R	B2R	180139-181653	504	100	99.8	99.2	99.2	99.2	100	100	100	100	100
	AKMV193	Ankyrin	198	B3R	B4R	181885-183555	556	100	100	99.5	99.3	99.3	100	100	100	100	100
	AKMV194	EEV type-1 membrane glycoprotein	199	B4R	B5R	183659-184612	317	100	100	99.7	99.7	99.7	100	100	100	100	100
	AKMV195	Ankyrin	200	B5R	B6R	184701-185231	176	100	100	97.7	97.7	97.7	100	100	101	101	101
	AKMV196	Virulence, ER resident	201	B6R	B7R	185270-185815	181	100	99.4	99.4	99.4	99.4	100	100	100	100	100
	AKMV197	Soluble interferon-gamma receptor-like protein	202	B7R	B8R	185893-186693	266	100	100	97.7	97.7	97.7	100	100	100	100	100
	AKMV198	Virulence factor	203	B8R	B9R	186835-187581	248	100	100	99.6	98	98	100	100	98.8	100	100
	AKMV199	Kelch-like protein	204	B9R	B10R	187715-189220	501	100	100	99.8	97.8	97.8	100	100	100	100	100
	AKMV200	-	205	B10R	B11R	189291-189578	95	100	100	100	97	97	100	100	97.9	93.7	93.7
	AKMV201	Ser/Thr Kinase	206	B11R	B12R	189645-190502	285	100	100	99.6	99.6	99.6	100	100	100	100	100
	AKMV202	Serpin	207	B12R	K2L	190593-191627	344	100	100	98.8	99.1	99.1	100	100	100	100	100
	AKMV203	-	208	B13R	C16L	191754-192203	149	100	100	99.3	100	100	100	100	100	100	100
	AKMV204	IL-1β receptor	209	B14R	B15.5R	192290-193276	328	100	100	98.5	98.8	98.8	100	100	100	100	100
	AKMV205	IL-1β receptor	210	B15L	B17L	194353-193331	340	100	100	98.5	98.2	98.2	100	100	100	100	100
	AKMV206	Ankyrin	211	B16R	B18R	194443-196230	595	100	100	99.3	99.3	99.3	100	100	100	98.5	98.5
	AKMV207	IFN-α/β binding protein	212	B17R	B19R	196253-197359	368	100	100	99.5	99.7	99.7	100	100	100	100	100
	AKMV208	Ankyrin	213	B18R	B20R	197440-199833	797	100	100	99	97.6	97.6	100	100	100	100	100
	AKMV209	Kelch-like protein	215	B19R	-	199940-201619	559	100	100	99.3	99.1	99.1	100	100	100	100	100
	AKMV210	Serpin	217	B20R	K2L	201879-203009	376	100	100	100	100	100	100	100	100	100	100
	AKMV211	ORF doesn't exist; missing ATG															
	AKMV212	Surface glycoprotein	219	B22R	-	204013-209790	1925	100	100	99.2	99.5	99.5	100	100	100	100	100
	AKMV213	Ankyrin	220	K1R	C19L	210043-211785	580	100	99.8	99.3	98.7	98.7	100	100	100	26	26
	AKMV214	TNF-alpha receptor	-	K3R	-	212581-213072	163	100	100	98.8	98.8	98.8	100	100	100	100	100
ITR region	AKMV215	-	222	I1R	C16L	213302-213763	153	100	100	98	98.7	98.7	100	100	100	100	100
	AKMV216	Ankyrin	223	I2R	C17L	213948-215963	671	100	100	98.1	98.8	98.8	100	100	100	100	100
	AKMV217	-	224	-	-	216082-216216	44	100	100	100	100	100	100	100	84.1	84.1	84.1
	AKMV218	Ankyrin	225	I3R	C19L	216228-217997	589	100	100	99.3	99.3	99.3	100	100	100	100	100
	AKMV219	TNF receptor (CrmB)	226	I4R	C22L	218084-219130	348	100	100	99.4	99.7	99.7	100	100	100	100	100
	AKMV220	Chemokine binding protein	227	I5R	C23L	219249-220001	250	100	100	98.8	98.4	98.4	100	100	100	100	100
